# Asthma prevalence and risk factors in early-onset atopic dermatitis using Korean National Health Insurance Service data

**DOI:** 10.1038/s41598-026-38149-8

**Published:** 2026-04-14

**Authors:** Jiwon Kim, Minyoung Jung, Ji Young Lee, Yechan Kyung, Kwanghoon Kim, Hoseob Kim, Mina Kim, Minji Kim

**Affiliations:** 1 Department of Pediatrics, Dankook University, Dankook University Hospital, Cheonan, Korea; 2 Department of Pediatrics, Samsung Medical Center, Sungkyunkwan University School of Medicine, Seoul, Korea; 3 Department of Pediatrics, Chuncheon Sacred Heart Hospital, Hallym University School of Medicine, Chuncheon, Korea; 4 Department of Pediatrics, Samsung Changwon Hospital, Sungkyunkwan University School of Medicine, Changwon, Korea; 5 Department of Pediatrics, Nowon Eulji Medical Center, Eulji University School of Medicine, Seoul, Korea; 6 Department of Data Science, Hanmi Pharm. Co., Ltd., Seoul, Korea; 7 Department of Pediatrics, College of Medicine, Chungnam National University, Chungnam National University Sejong Hospital, Sejong, Korea

**Keywords:** Atopic dermatitis, Asthma, Early-onset, Pediatric, Risk factors, Allergic march, Diseases, Health care, Medical research, Risk factors

## Abstract

Early-onset atopic dermatitis (AD) is a common pediatric allergic disease and a potential precursor of asthma. However, population-based evidence on long-term asthma risk following early AD is scarce. This study assessed age-specific asthma prevalence and risk factors in children diagnosed with AD before age 2 using Korean National Health Insurance Service data. In this retrospective cohort, children with AD diagnoses (ICD-10: L20) at least twice before age 2 between 2008 and 2017 were included. Asthma was defined by diagnosis (ICD-10: J45, J46) and concurrent prescription of asthma medication. Patients were followed until December 2022. Standardized prevalence ratios (SPRs) were calculated relative to the general population, and multivariate logistic regression identified risk factors. Of 1,647,199 children with early-onset AD, 1,161,172 were analyzed. Asthma prevalence peaked at age 3 (29.0%) and declined to 0.5% by age 15, remaining significantly higher than in the general population. Increased risk factors included male sex, preterm birth, diagnosis before 2010, persistent AD, food allergy, and early rhinovirus or RSV infection. Diagnosis after age 1 and higher socioeconomic status were linked to lower risk. Early-onset AD is strongly associated with later asthma, highlighting the need for early identification and targeted prevention.

**Supplementary Information:**

The online version contains supplementary material available at 10.1038/s41598-026-38149-8.

## Introduction

Atopic dermatitis (AD) is one of the most common allergic diseases in children and is characterized by a chronic inflammatory skin disease^1^. In the International Study of Asthma and Allergy in Children, the prevalence of AD in children varies widely, ranging from 0.3% to 20.5% in 56 countries^2^. AD typically peaks in incidence during the first 2 years of life^1,3^. In Korea, prevalence is also highest among children under 2 years, ranging from 5.1 to 20.1% according to the Korean National Health Insurance Service (KNHIS)^3^.

AD in infants and young children causes substantial social burden and patient distress; however, allergic diseases that begin with AD are known to progress to other allergic conditions due to allergic march^4^. The allergic march refers to a well-known phenomenon in which patients with AD often progress to other allergic diseases, such as allergic rhinitis (AR) and asthma, more frequently than individuals in the general population^4,5^. Asthma is one of the leading allergic diseases, with a high incidence in children. It is an important chronic respiratory disease in children with a high socioeconomic burden^6,7^. However, allergic march progression is not universal, and the number of patients who develop it remains controversial. In Taiwan, 21.6% of patients with AD before the age of 3 developed asthma after 8 years of follow-up, while in Japan, 35% developed asthma after 4 years of follow-up^8,9^. However, not all patients with AD progress to asthma during early childhood, and studies suggest that asthma development is more likely in specific phenotypes of AD, particularly those accompanied by IgE sensitization or by early-onset and severe AD^10^. Understanding which risk factors predispose patients to allergic progression is critical for developing effective prevention and treatment strategies.

The KNHIS database is a comprehensive framework that covers the entire population of South Korea and provides a rich source of data for health-related research^11^. The system plays a fundamental role in understanding health outcomes, healthcare expenditures, and the utilization of healthcare services across different demographics and conditions^12,13^. Data from the NHIS span from birth through the cycle course, and the comprehensive nature of the database allows for longitudinal studies that can provide insights into health trends, disease prevalence, and outcomes^13,14^. This study aimed to investigate the prevalence of asthma in patients with early-onset AD and to determine whether it is higher than that in the general population. Additionally, we evaluated the prevalence of asthma and its risk factors over the life course of patients with early-onset AD using data from the NHIS database.

## Methods

### Data sources and ethical approval

The KNHIS program covers more than 98% of the population, providing a comprehensive dataset that includes nearly the entire nation^15–17^. This extensive data has been used widely in clinical research as a valuable source of real-world evidence^16^. The NHIS database encompasses a wide range of information, including demographic details, medications, and diagnostic codes. Demographic data include patient age, sex, socioeconomic status, and residence^18,19^. Diagnoses were coded using the International Classification of Diseases (ICD)-10, which also guides data extraction for research purposes. The study was conducted in accordance with the Declaration of Helsinki and approved by the Institutional Review Board of Chungnam National University Sejong Hospital, Sejong, Republic of Korea (IRB Number: 2023-07-001-002). Given the descriptive nature of the study and the use of public data without personal identifiers, the board waived the requirement for informed consent.

## Study population and covariates

The study included children diagnosed with AD (ICD code: L20) aged 0–2 years on at least two occasions between 2008 and 2017. Patients with congenital disorders and chromosomal abnormalities (ICD-10: Q00-Q99) were excluded from the study. Asthma was defined as having a diagnosis code (ICD-10: J45, J46) and being prescribed asthma medication. The enrolled children with AD were followed up until December 2022, using the NHIS data. Asthma medications included inhaled corticosteroids (ICS), long-acting β2 agonists (LABA), ICS/LABAs, short-acting β_2_ agonists (SABA), leukotriene receptor antagonists, anticholinergics, and xanthines (Suppl. Table 1).

This longitudinal study determined the prevalence of asthma and its risk factors in children with AD under 2 years of age. To identify the relevant risk factors for asthma, we analyzed data on rhinovirus infection (ICD-10: J206), respiratory syncytial virus (RSV) infection (J205, J210, J121), and food allergy (T78.1) occurring before age 2. Patients with AD were categorized into persistent, intermittent, and remitted AD groups according to whether they had a hospital visit every year since the age of 2. The data contain information about the participants’ household income classes (1st–20th quantiles), which the NHIS uses to calculate insurance premiums. We divided household income into five levels (Q1, 1–4; Q2, 5–8; Q3, 9–12; Q4, 13–16; Q5, 17–20).

### Statistical analysis

The baseline demographic and clinical characteristics were summarized using descriptive statistics. Categorical variables are presented as frequencies and percentages. To evaluate the relative prevalence of asthma in patients with early-onset AD compared to the general population, standardized prevalence ratios (SPRs) were calculated to compare the prevalence of asthma among individuals with AD to that in the general Korean population. Sex- and year–specific prevalence rates were obtained from a previous study^20^. The SPR was calculated by dividing the observed number of asthma cases in the AD group by the expected number based on national sex-specific rates. The 95% confidence interval (CI) for SPR was calculated using the Poisson distribution. An SPR greater than 1 indicates a higher prevalence of asthma in the AD group than in the general population. To identify independent risk factors associated with asthma among patients with early-onset AD, we performed a multivariable logistic regression analysis. The variables included in the model were selected based on their clinical relevance and statistical significance in univariate analyses. The adjusted odds ratios (aORs) with 95% CIs were calculated. Statistical significance was defined as a two-sided p-value of < 0.05. All analyses were conducted using SAS version 9.4 (SAS Institute Inc., Cary, NC, USA).

## Results

**Fig. 1:**
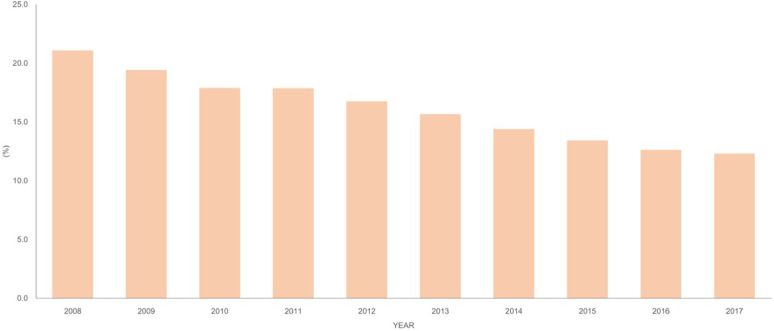
Prevalence of atopic dermatitis in children under 2 years of age in South Korea.

From 2008 to 2017, a total of 1,647,199 patients aged 2 years or younger were diagnosed with AD as a primary diagnosis at least twice, of which 1,161,172 were used in the analysis, excluding those diagnosed with congenital diseases and chromosomal abnormalities or those diagnosed with asthma prior to AD (Supp. Figure 1). As a percentage of the total population, AD in children aged 2 years and younger was 21.1% in 2008, 19.4% in 2009, 17.9% in 2010, 17.9% in 2011, 16.7% in 2012, 15.7% in 2013, 14.4% in 2014, 13.4% in 2015, 12.6% in 2016, and 12.3% in 2017 (Fig. 1). Demographic characteristics are shown in Table 1. A total of 51.5% of patients with AD were male. AD was diagnosed in 44.0% of children aged < 1 year, 41.7% of children aged 0–1 year, and 14.4% of children aged 1–2 years. Overall, 59.5% of the patients were in remission after 2 years of age, 40.0% required intermittent treatment afterwards, and 0.5% required continuous treatment during follow-up. Overall, 21.6% of patients with AD lived in the capital city of Seoul, 22.1% in metropolitan areas, and 56.3% in other rural areas. Comorbidities included food allergy in 0.98% of cases, rhinovirus infection in 0.69%, and RSV infection in 2.47% of cases under 2 years of age.

**Table 1:** Demographic characteristics of the patients with early-onset atopic dermatitis (AD) (*N* = 1,161,172).

Variable		Total		Only AD		AD to asthma		*P*- value
		*N*	%	*N*	%	*N*	%	
Total		1,161,172		560,534	100.00	600,638	100.00	
Sex	Male	597,427	51.45	275,691	49.18	321,736	53.57	< 0.0001
	Female	563,745	48.55	284,843	50.82	278,902	46.43	
Preterm birth		2,8348	2.44	12,983	2.32	15,365	2.56	
Age at diagnosis (years)	0	510,377	43.95	230,687	41.15	279,690	46.57	< 0.0001
	0–1	483,832	41.67	233,554	41.67	250,278	41.67	
	1–2	166,963	14.38	96,293	17.18	70,670	11.77	
Persistence of AD	Persistent	5,924	0.51	1,980	0.35	3,944	0.66	< 0.0001
	Intermittent	464,753	40.02	208,983	37.28	255,770	42.58	
	Remission	690,495	59.47	349,571	62.36	340,924	56.76	
Region of residence	Seoul	251,054	21.62	124,544	22.22	126,510	21.06	< 0.0001
	Metropolitan	256,676	22.10	123,274	21.99	133,402	22.21	
	Rural area	653,442	56.27	312,716	55.79	340,726	56.73	
Income	Q1 (lowest)	96,358	8.30	47,085	8.40	49,273	8.20	< 0.0001
	Q2	116,994	10.08	54,149	9.66	62,845	10.46	
	Q3	281,470	24.24	132,208	23.59	149,262	24.85	
	Q4	368,836	31.76	178,385	31.82	190,451	31.71	
	Q5 (highest)	224,292	19.32	113,666	20.28	110,626	18.42	
Enrolled Year	2008	156,154	13.45	52,896	9.44	103,258	17.19	< 0.0001
	2009	138,931	11.96	50,960	9.09	87,971	14.65	
	2010	128,429	11.06	51,528	9.19	76,901	12.80	
	2011	130,925	11.28	57,918	10.33	73,007	12.15	
	2012	124,619	10.73	60,798	10.85	63,821	10.63	
	2013	115,025	9.91	60,843	10.85	54,182	9.02	
	2014	104,406	8.99	58,399	10.42	46,007	7.66	
	2015	95,845	8.25	57,101	10.19	38,744	6.45	
	2016	86,577	7.46	55,103	9.83	31,474	5.24	
	2017	80,261	6.91	54,988	9.81	25,273	4.21	
Personal medical history (before 2 years old)								
Food allergy		11,385	0.98	5,462	0.97	5,923	0.99	0.5229
Rhinovirus infection		8,039	0.69	3,586	0.64	4,453	0.74	< 0.0001
Respiratory syncytial virus infection		28,675	2.47	10,651	1.90	18,024	3.00	< 0.0001

Values are presented as numbers (%).

**Fig. 2:**
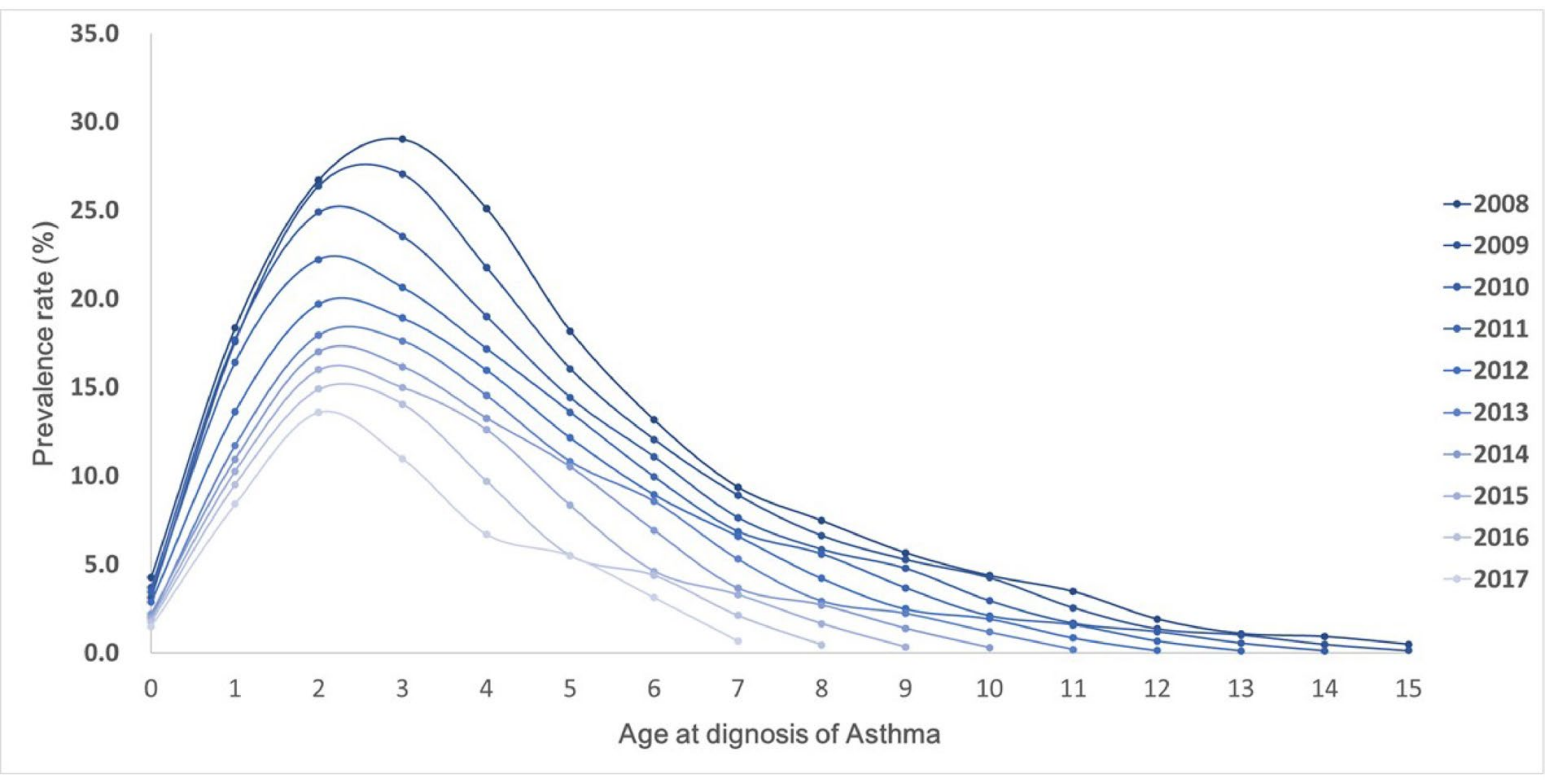
Trends in age-specific asthma prevalence in children with atopic dermatitis between 2008 and 2017.

Patients with AD were followed up until 2022 to determine the prevalence of asthma at each age point (Fig. 2). The prevalence of asthma in patients with AD enrolled in 2008 was 29.0% at 3 years of age and gradually decreased thereafter, reaching 0.5% at 15 years of age. In 2017, the prevalence of asthma in patients enrolled was 11.0% at 3 years of age and decreased to 3.1% at 6 years of age.

The SPR of asthma in patients with early-onset AD was compared with that of the general population, showing the following pattern: SPR: 145.7 (95% CI: 144.1–147.3) in children aged 1 month to 2 years, SPR: 231.7 (95% CI: 230.2–233.2) in 2–4 years, SPR: 87.9 (95% CI: 87.3–88.5) in 4–6 years, SPR: 80.9 (95% CI: 80.4–81.4) in 6–12 years, and SPR: 2.0 (95% CI: 1.9–2.1) in 12–15 years.

**Fig. 3:**
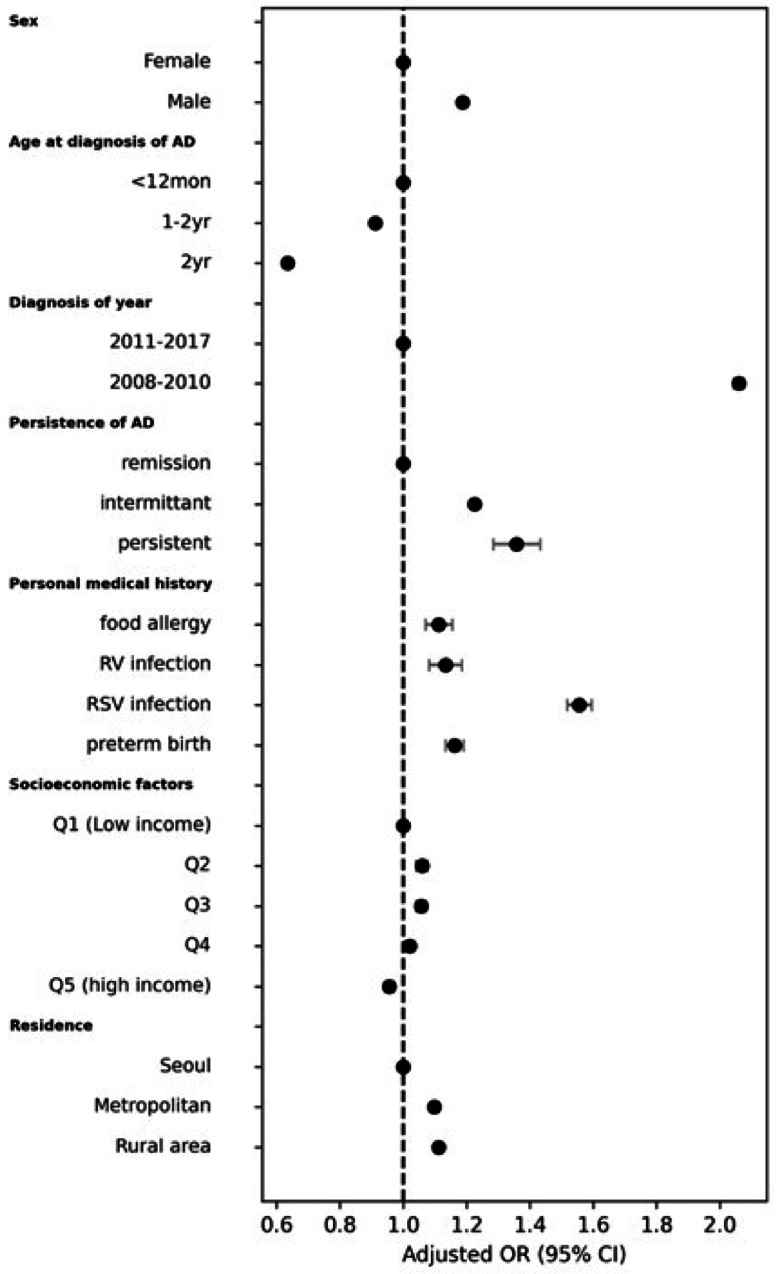
Risk of asthma in patients with early-onset atopic dermatitis.

The risk factors for asthma development in patients with early-onset AD are shown in Fig. 3 (Table 2). Significant predictors of asthma included being male (aOR, 1.187; 95% CI, 1.178–1.196) and preterm birth (aOR, 1.162; 95% CI, 1.134–1.19). The risk of asthma was also higher in patients diagnosed with AD before 2010 compared with those diagnosed during 2011–2017 (aOR, 2.059; 95% CI, 2.043–2.075). Patients diagnosed with AD at ages 1–2 years had a lower risk of developing asthma than those diagnosed at age 0 (aOR, 0.634; 95% CI, 0.626–0.641). Patients who had persistent AD throughout their lives had an increased risk of asthma compared to those who experienced remission after age 2 (aOR, 1.357; 95% CI, 1.284–1.433). When comparing the risk of asthma prevalence in patients with higher incomes compared to those with lower incomes, a lower prevalence of asthma was observed in the highest income group (aOR, 0.956; 95% CI, 0.941–0.971). Compared to patients living in the capital city of Seoul, the risk of developing asthma was higher for those living in metropolitan areas (aOR, 1.098; 95% CI, 1.086–1.111) and rural areas (aOR, 1.111; 95% CI, 1.101–1.122). Additional risk factors included food allergy (aOR, 1.112; 95% CI, 1.071–1.155), rhinovirus infection (aOR, 1.133; 95% CI, 1.082–1.185), and RS virus infection (aOR, 1.556; 95% CI, 1.518–1.595) before age 2.

**Table 2:** Risk of asthma in patients with early-onset atopic dermatitis (AD).

Variable		Asthma	
	Reference	Crude OR	Adjusted OR
Male	Female	1.192(1.183-1.2)	1.187(1.178–1.196)
Preterm birth	Full-term birth	1.107(1.081–1.133)	1.162(1.134–1.19)
Diagnosis of the year (2008–2010)	2011–2017	2.103(2.086–2.119)	2.059(2.043–2.075)
Age of diagnosis (year)			
0–1	O year of AD	0.884(0.877–0.891)	0.911(0.904–0.919)
1–2		0.605(0.599–0.612)	0.634(0.626–0.641)
Persistence of AD			
Persistent AD	Remission	2.042(1.935–2.156)	1.357(1.284–1.433)
Intermittent AD		1.255(1.246–1.264)	1.225(1.215–1.234)
Socioeconomic factors			
Income			
Q2	Q1 (Low income)	1.109(1.09–1.128)	1.059(1.04–1.077)
Q3		1.079(1.063–1.095)	1.056(1.04–1.072)
Q4		1.02(1.006–1.035)	1.021(1.006–1.035)
Q5 (high income)		0.93(0.916–0.944)	0.956(0.941–0.971)
Residence			
Metropolitan	Seoul (Capital city)	1.065(1.054–1.077)	1.098(1.086–1.111)
Rural area		1.073(1.063–1.083)	1.111(1.101–1.122)
Personal medical history (before 2 years old)			
Rhinovirus infection	None	1.159(1.109–1.212)	1.133(1.082–1.185)
RSV infection	None	1.597(1.559–1.636)	1.556(1.518–1.595)
Food allergy	None	1.012(0.975–1.05)	1.112(1.071–1.155)

We further analyzed age-stratified risk factors for asthma development in patients with early AD (Suppl. Figure 2, Table 3). The multivariate logistic regression analysis revealed that male sex and preterm birth were associated with an increased risk of asthma across multiple age groups. Notably, persistent and intermittent AD were consistently strong predictors of asthma development in all age groups. The aOR for asthma in patients with persistent AD increased with age, from 1.39 (95% CI: 1.32–1.46) in 0–2 years to 8.14 (95% CI: 6.99–9.47) in 13–15 years. Patients living outside of Seoul; both in metropolitan and other provincial areas generally showed higher odds of developing asthma compared to those residing in Seoul, particularly in younger age groups: aOR 1.13 (95% CI: 1.12–1.15) for metropolitan areas and 1.17 (95% CI: 1.16–1.18) for rural area compared to capital city residence. However, this regional disparity in asthma risk diminished with age and even reversed in adolescents aged 13–15 years, with aORs below 1.0 in non-capital city regions.

**Table 3:** Age-stratified risk factors for asthma development in patients with early atopic dermatitis (AD).

Variable	Reference	Asthma									
		0–2yrs		3–6yrs		7–9yrs		10–12yrs		13–15yrs	
		Crude OR	Adjusted OR	Crude OR	Adjusted OR	Crude OR	Adjusted OR	Crude OR	Adjusted OR	Crude OR	Adjusted OR
Male	Female	1.25 (1.24–1.26)	1.25 (1.24–1.26)	1.11 (1.1–1.12)	1.11 (1.1–1.12)	1.2 (1.19–1.21)	1.21 (1.19–1.22)	1.38 (1.35–1.41)	1.39 (1.36–1.42)	1.32 (1.26–1.39)	1.34 (1.27–1.41)
Preterm	Non	1.07 (1.04–1.1)	1.07 (1.04–1.09)	1.12 (1.09–1.15)	1.12 (1.09–1.15)	1.08 (1.04–1.12)	1.09 (1.04–1.13)	1.09 (1.02–1.16)	1.1 (1.03–1.17)	0.9 (0.76–1.07)	0.91 (0.77–1.08)
Persistent AD	Remission	1.37 (1.3–1.45)	1.39 (1.31–1.46)	2.0 (1.9–2.1)	2.01 (1.91–2.11)	2.75 (2.58–2.94)	2.77 (2.6–2.95)	4.53 (4.16–4.93)	4.57 (4.2–4.97)	8.15 (7.0–9.49)	8.13 (6.99–9.47)
Intermittent AD		1.09 (1.08–1.1)	1.1 (1.09–1.11)	1.29 (1.28–1.3)	1.29 (1.28–1.3)	1.42 (1.4–1.44)	1.43 (1.41–1.44)	1.56 (1.53–1.6)	1.57 (1.54–1.61)	1.76 (1.67–1.85)	1.76 (1.67–1.85)
Income											
Q2	Q1 (Low income)	1.07 (1.05–1.09)	1.07 (1.05–1.09)	1.08 (1.06–1.1)	1.08 (1.06–1.1)	1.12 (1.09–1.16)	1.12 (1.09–1.15)	1.17 (1.12–1.23)	1.17 (1.11–1.22)	1.18 (1.06–1.32)	1.17 (1.05–1.31)
Q3		1.04 (1.03–1.06)	1.04 (1.03–1.06)	1.07 (1.05–1.09)	1.07 (1.05–1.09)	1.05 (1.02–1.07)	1.05 (1.02–1.07)	1.06 (1.01–1.1)	1.05 (1.01–1.1)	0.99 (0.9–1.09)	0.99 (0.9–1.09)
Q4		0.98 (0.96–0.99)	0.99 (0.97–1.0)	1.03 (1.01–1.04)	1.03 (1.02–1.05)	0.99 (0.97–1.02)	0.99 (0.97–1.02)	0.98 (0.94–1.02)	0.98 (0.94–1.02)	0.86 (0.78–0.94)	0.85 (0.78–0.94)
Q5 (high income)		0.86 (0.85–0.88)	0.88 (0.86–0.89)	0.96 (0.94–0.97)	0.96 (0.95–0.98)	0.92 (0.89–0.94)	0.92 (0.89–0.94)	0.89 (0.85–0.93)	0.88 (0.84–0.92)	0.83 (0.75–0.93)	0.82 (0.74–0.91)
Residence											
Metropolitan	Seoul	1.15 (1.13–1.16)	1.13 (1.12–1.15)	1.04 (1.02–1.05)	1.03 (1.02–1.05)	0.95 (0.93–0.96)	0.94 (0.93–0.96)	0.91 (0.88–0.93)	0.9 (0.87–0.93)	0.79 (0.73–0.85)	0.79 (0.73–0.85)
Rural area		1.18 (1.17–1.19)	1.17 (1.16–1.18)	1.04 (1.03–1.05)	1.05 (1.04–1.06)	1.0 (0.98–1.02)	1.0 (0.98–1.01)	0.94 (0.92–0.97)	0.94 (0.92–0.96)	0.88 (0.83–0.93)	0.88 (0.82–0.93)
Personal medical history (before 2 years old)											
FA	Non-FA	1.08 (1.04–1.12)	1.06 (1.02–1.1)	-	-	-	-	-	-	-	-
Rhinovirus infection	No	1.21 (1.15–1.26)	1.04 (0.99–1.09)	-	-	-	-	-	-	-	-
RSV infection	No	1.77 (1.73–1.81)	1.76 (1.72–1.8)	-	-	-	-	-	-	-	-

yrs, years, OR, odds ratios, FA, food allergy.

## Discussion

This study aimed to investigate the age-specific prevalence of asthma among patients with early-onset AD and identify the associated risk factors. We utilized longitudinal data from the Korean NHIS to track patients diagnosed with AD before the age of 2, registered from 2008 to 2017, until 2022. Asthma prevalence was highest in early childhood and declined progressively into adolescence, underscoring a critical window for intervention. Several factors were associated with an increased risk of asthma development in patients with AD, notably male sex, preterm birth, very early onset (before 1 year), persistent AD, low socioeconomic status, and respiratory viral infections.

We compared the prevalence of asthma in patients with early-onset AD with the previously reported asthma prevalence in the general population^20^. Our analysis revealed that the risk of asthma in the AD cohort was significantly higher than that in the general population across all age groups. Although the SPR gradually decreased with age, the risk remained elevated, showing approximately a two-fold increase even at 15 years of age. These findings provide objective population-based evidence that children with early-onset AD have a substantially higher risk of developing asthma than the general population.

Several studies have identified risk factors for the progression of asthma in patients with AD, including genetic, immunological, and environmental factors^21^. Early-onset AD has been consistently associated with an elevated risk of asthma development^22,23^. Our study also demonstrated that patients diagnosed with AD before the age of 1 were more likely to develop asthma. The severity and persistence of AD also play crucial roles; patients with persistent or severe forms of AD exhibit a significantly higher incidence of asthma than those in remission^24,25^. In this study, AD severity was estimated based on the presence of recurrent outpatient clinic revisits. During the follow-up, patients who requiring at least one annual clinic visit for ongoing AD management demonstrated a higher risk of asthma compared with those who achieved remission after age 2.

Socioeconomic and environmental factors also influence the risk of asthma. Low-income status and rural residence were both associated with an increased risk of developing asthma. Ineffective management of AD has been implicated as a major contributor to asthma progression^26^. Enhancing skin barrier function through appropriate treatment may reduce allergen penetration and systemic inflammation, thereby mitigating the risk of asthma development^27^. Reduced access to healthcare resources and suboptimal disease management may exacerbate asthma progression risks. Public health interventions targeting socioeconomically disadvantaged populations are critical in addressing disparities in disease outcomes. Interestingly, when asthma prevalence was categorized by age group, living in the capital city was associated with a decreased risk for children up to the age of 6 years but was associated with an increased risk for children aged 7 years and older. Industrialized environments in urban areas may exacerbate asthma risk as children grow older, a hypothesis supported by previous studies on environmental triggers of asthma^28,29^.

Despite its strengths, this study has limitations inherent in its reliance on administrative data from the Korean NHIS database. The reliance on administrative data may have introduced bias in clinical diagnoses, as the data were collected primarily for billing purposes. While age-stratified analyses capture real-world diagnostic patterns, claims-based data do not permit definitive phenotypic differentiation between transient wheeze and asthma, particularly in early childhood. In addition, the lack of genetic or environmental exposure data limits our ability to comprehensively explore the underlying mechanisms. Nevertheless, the large sample size and long follow-up period provided valuable insights into the long-term outcomes of pediatric patients with AD. These findings highlight the complex interplay between genetic predisposition, environmental exposure, and socioeconomic factors in the progression of AD to asthma. Future research should focus on elucidating the mechanisms underlying these associations and exploring strategies to interrupt the atopic march.

In conclusion, children with early-onset AD face a disproportionately high risk of developing asthma, particularly in early childhood. Male sex, preterm birth, persistent AD, and socioeconomic disadvantage exacerbate this risk. These findings underscore the importance of targeted interventions for improving disease management and reducing disparities in healthcare access.

## Supplementary Information

Below is the link to the electronic supplementary material.

Supplementary Material 1

Supplementary Material 2

Supplementary Material 3

Supplementary Material 4

## Data Availability

Although data are accessible from the National Health Insurance (NHI) database, access to the data used in this study is only provided to researchers who have applied for and obtained permission. Additional information is available on the online homepage of the NHI Sharing Service (https://nhiss.nhis.or.kr).
