# Supplementary figures and images for: Asthma prevalence and risk factors in early-onset atopic dermatitis using Korean National Health Insurance Service data
Source: Sci Rep. 2026 Apr 14;16:12267. doi: 10.1038/s41598-026-38149-8 (PMC13079820; doi:10.1038/s41598-026-38149-8)

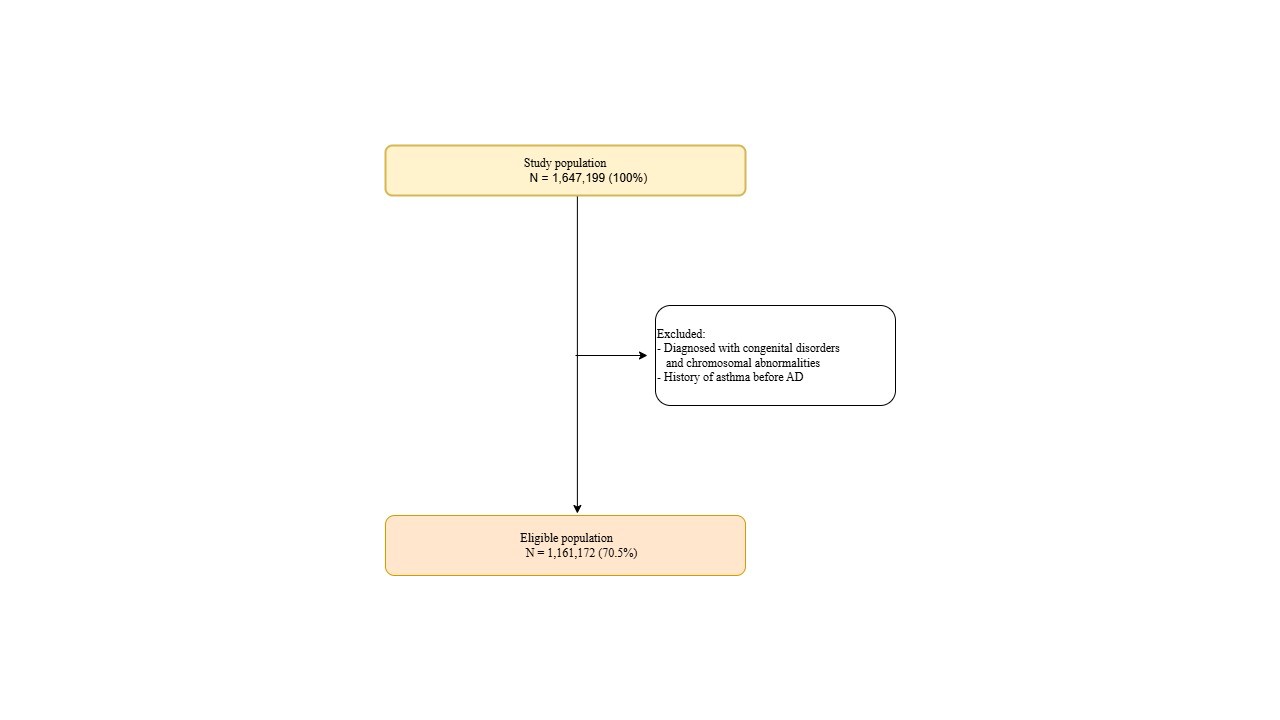

Supplement: Supplementary file 1 — Supplementary Material 1 [file 41598_2026_38149_MOESM1_ESM.jpg]

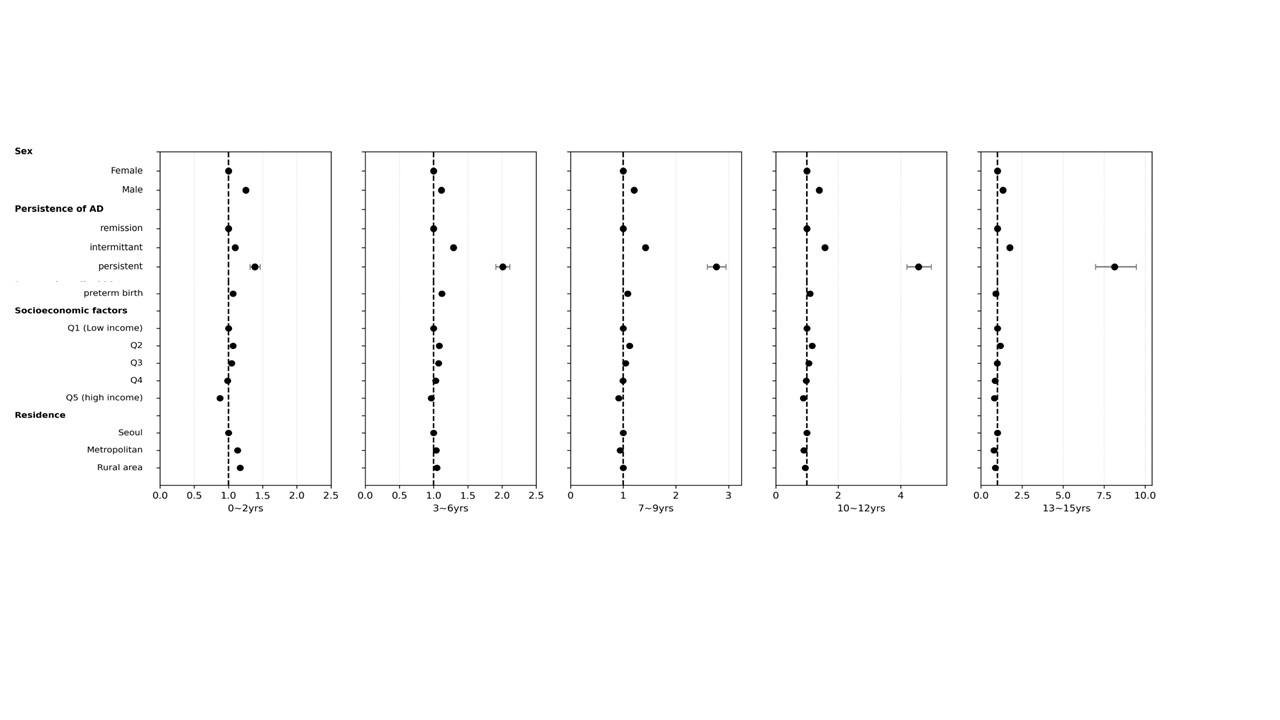

Supplement: Supplementary file 2 — Supplementary Material 2 [file 41598_2026_38149_MOESM2_ESM.jpg]
