# Supplementary material for: Asthma prevalence and risk factors in early-onset atopic dermatitis using Korean National Health Insurance Service data
Source: Sci Rep. 2026 Apr 14;16:12267. doi: 10.1038/s41598-026-38149-8 (PMC13079820; doi:10.1038/s41598-026-38149-8)
Supplement: Supplementary file 3 — Supplementary Material 3 [file 41598_2026_38149_MOESM3_ESM.docx]

Supplement Figure 1. Flow chart of participant eligibility and inclusion

Supplement Figure 2. Age-stratified risk factors for asthma in patients with early-onset atopic dermatitis
